# Social camouflaging of autistic traits is associated with more severe symptoms among subjects with feeding and eating disorders

**DOI:** 10.1007/s40519-025-01785-6

**Published:** 2025-10-15

**Authors:** Barbara Carpita, Benedetta Nardi, Stefano Pini, Francesca Parri, Paola Perrone, Cristiana Pronestì, Federico Giovannoni, Gerardo Russomanno, Chiara Bonelli, Gabriele Massimetti, Ivan Mirko Cremone, Andrea Fiorillo, Liliana Dell’Osso

**Affiliations:** 1 Department of Clinical and Experimental Medicine, Section of Psychiatry, University of Pisa, 67 Via Roma 4, 56126 Pisa, Italy; 2 Department of Biotechnology, Chemistry and Pharmacy, University of Siena, Siena, Italy; 3 Department of Psychiatry, University of Campania “Luigi Vanvitelli”, Naples, Italy

**Keywords:** Camouflaging, Autistic traits, Feeding and eating disorders, Anorexia, Bulimia, Binge eating disorder

## Abstract

**Purpose:**

Given the high prevalence of autistic traits among individuals with eating disorders (EDs), this study investigates the relationship between social camouflaging and eating disorder symptoms. It specifically examines how camouflaging behaviors may influence the manifestation and severity of disordered eating.

**Methods:**

A total of 70 patients with EDs and 50 healthy controls (HCs) were assessed using the Camouflaging Autistic Traits Questionnaire (CAT-Q) and the Eating Disorder Inventory-2 (EDI-2). Independent samples *t* tests were used to compare CAT-Q scores between groups. ANOVA followed by Bonferroni post-hoc tests examined differences across EED subtypes. Pearson correlation analyses assessed associations between CAT-Q and EDI-2 scores. Finally, a linear regression model was used to evaluate whether camouflaging (CAT-Q total score) significantly predicted eating disorder symptom severity (EDI-2 total score).

**Results:**

ED patients scored significantly higher than HCs across all CAT-Q domains and on the total score (all *p* < .001). No significant differences in camouflaging scores were observed among the different ED subtypes. CAT-Q domain and total scores were significantly positively correlated with all EDI-2 domains, with few exceptions. Linear regression analysis indicated that CAT-Q total score was a significant predictor of EDI-2 total score (*β* = .728, *p* < .001).

**Conclusions:**

Our findings reinforce the notion that social camouflaging, often used as a coping strategy, is associated with the presence and severity of eating disorder symptoms. Overall, the study underscores the complex interplay between autistic traits and disordered eating, highlighting the importance of further research into this connection.

**Level of evidence:**

Level III: Evidence obtained from well-designed cohort or case–control analytic studies.

## Introduction

Eating disorders (EDs) are complex and severe psychiatric conditions characterized by abnormal eating behaviors and dysfunctional weight-control practices [1]. These disorders are not merely lifestyle choices but are often rooted in profound psychological distress and disturbed attitudes toward food, body shape, and weight. Central to their development and persistence are cognitive and emotional distortions surrounding eating and self-perception, which contribute significantly to impaired daily functioning and serious medical complications [2].

The most recent editions of the Diagnostic and Statistical Manual of Mental Disorders (DSM-5-TR) and the International Classification of Diseases (ICD-11) recognize six core EDs: anorexia nervosa (AN), bulimia nervosa (BN), binge eating disorder (BED), as well as pica, rumination disorder, and avoidant/restrictive food intake disorder (ARFID) [3, 4]. While AN, BN, and BED are well-established diagnoses with substantial clinical literature, the inclusion of disorders, such as pica and rumination disorder—traditionally associated with pediatric populations—reflects a growing understanding of the diverse presentations of EDs across the lifespan. The DSM-5-TR includes important refinements, including severity specifiers, remission criteria, and diagnostic subtypes, which enhance clinical utility. Although the DSM-5-TR generally applies more stringent criteria than the ICD-11, both systems aim to improve diagnostic precision and treatment relevance [5].

EDs commonly emerge during adolescence, a crucial period of neurobiological and psychological development; however, the exact age of onset varies slightly amongst them. Moreover, though historically viewed as predominantly affecting females, new evidences indicate that also a sizable percentage of men also experience EDs [6]. Many individuals with significant eating pathology do not meet full diagnostic criteria but nonetheless experience considerable distress and impairment. This has led to growing interest in a dimensional or spectrum-based approach, which recognizes subthreshold conditions and shared psychopathological traits that cut across diagnostic boundaries. Such an approach may offer a more comprehensive framework for understanding the heterogeneity and complexity of EDs in both clinical and community populations.

In recent years, there has been growing recognition of the complex relationship between autism spectrum disorder (ASD) and EDs. ASD is a neurodevelopmental condition marked by persistent difficulties in social communication and interaction, alongside restricted, repetitive patterns of behavior, interests, or activities [3]. The clinical presentation of ASD ranges through a broad continuum—from individuals with significant intellectual and language impairments to those with milder symptoms, average intelligence and language development, who maintain independence in daily living [3]. In addition, autism spectrum is understood not as a discrete diagnosis but as a spectrum of traits that exist dimensionally across the general population. These traits can range from subclinical or isolated characteristics observed in neurotypical individuals to more pronounced features meeting the criteria for a formal ASD diagnosis [7].

Although ED and ASD are classified as separate diagnostic conditions, a potential link between those two dimensions has increasingly been proposed, based on both shared familial patterns and overlapping behavioral traits [8–12]. Initially, a possible genetic link between ED and ASD was first proposed by Gillberg in 1983, based on his clinical observations. He noted that boys with autism often had relatives with ED and suggested that AN might represent a female manifestation of autism [13]. Over the past decade, numerous studies have found a high rate of autistic traits among individuals with ED [12, 14]. These individuals often show neuropsychological and socioemotional profiles that resemble those seen in autism [15–22], including difficulties with emotion recognition [23], empathy [24], and emotional awareness. Additional challenges include attentional control, perspective-taking, and theory of mind [25]. There is also evidence of shared neurocognitive traits and temperamental features between ED and ASD [26].

Moreover, a review by Westwood and Tchanturia [14] found that 20–30% of women receiving treatment for AN meet the clinical threshold for ASD. These autistic characteristics are often present even before the onset of restrictive eating behaviors [27] and can persist in those who have recovered from EDs [28]. Moreover, eating behaviors commonly observed in individuals with ASD—especially those involving social interactions during meals—are also prevalent in EDs and may remain after physical recovery [29]. The overlap in cognitive and social impairments across both conditions points to potential shared biological mechanisms [30], supporting the notion that ASD and ED symptoms often co-occur at a higher-than-expected rate. Interestingly, a longitudinal study conducted in 2018 tested whether prolonged malnutrition might trigger autistic traits, hypothesizing these traits would diminish with proper nutritional rehabilitation. However, findings indicated that both autistic traits and diagnoses remained stable after 12 months of treatment, underscoring the importance of identifying ASD early in individuals presenting with EDs [31]. Similarly, Brown and Stokes argued that ED’s clinicians are often the first to recognize signs of autism, particularly in female patients [32]. Their review emphasized the need for further exploration into the comorbidity, treatment considerations, and future research priorities related to ASD and EDs.

Moreover, in a mixed-method study involving autistic women, parents of adolescents with ASD, and healthcare professionals, Babb et al. discovered that all the women were diagnosed with autism only after beginning treatment for an eating disorder [33]. The average age of ED diagnosis was 17, while ASD diagnosis came later, at an average age of 29. Similarly, a qualitative study involving individuals with both ED and ASD revealed that ASD was often diagnosed during or after ED treatment, with an average diagnosis age of 23.5 [34]. Finally, recent genomic research using polygenic scores (PGS) has shown a notable genetic correlation between ASD and AN. Individuals with ASD or who had a sibling—particularly a full or maternal half sibling—with ASD were at increased risk of developing AN. Furthermore, elevated ASD-related polygenic scores were associated with a higher risk for AN [35]. These overlapping characteristics suggest that neurodevelopmental features may play a significant role in the development, presentation, and maintenance of disordered eating behaviors. According to this view, the restricted interests and repetitive behaviors characteristic of ASD may, in some females, become focused on food, body image, and dieting behaviors [36].

In addition to clinically diagnosed ED, individuals with ASD and those high in autistic traits frequently exhibit atypical eating patterns, such as food selectivity, food neophobia, and symptoms consistent with ARFID. These behaviors are often linked to sensory sensitivities, cognitive rigidity, and heightened anxiety around novel foods—features commonly associated with the autism spectrum. Studies have shown that food selectivity is significantly more prevalent in autistic individuals compared to neurotypical peers, and that these patterns may persist into adolescence and adulthood, impacting nutritional status and quality of life [37, 38].

One construct that has gained attention in this context is social camouflaging, a set of strategies used to mask or compensate for autistic traits to appear more neurotypical in social situations [39, 40]. While camouflaging behaviors are observed across sexes, they are believed to be more frequently and effectively employed by females [41–43]. This tendency may contribute to the underdiagnosis of ASD in women, as females with ASD often present with subtler social impairments and more developed adaptive coping mechanisms. As a result, they are less likely to meet the conventional diagnostic thresholds, contributing to the consistently reported male-to-female diagnostic ratio of approximately 3:1 [44]. Although camouflaging is often associated with better short-term social integration, it has also been linked to heightened psychological distress, identity confusion, delayed diagnoses and psychiatric comorbidities [39]. While much of the research on camouflaging has focused on individuals formally diagnosed with autism, emerging evidence suggests that these behaviors may also be prevalent in non-diagnosed populations who exhibit subclinical autistic traits [45, 46]. Intriguingly, a recent study highlighted how among subjects with ASD camouflaging behaviors may predict eating disorder symptomatology [47].

Given the high rates of autistic traits (and sometimes unrecognized ASD) among individuals with EDs the present study aims to explore the association between social camouflaging and eating disorder symptoms, with a particular focus on how camouflaging may contribute to the presentation and severity of disordered eating.

## Materials and methods

### Study sample and procedures

For this research, we recruited 70 consecutive patients diagnosed with a Feeding and Eating Disorder, and who met eligibility criteria, who were receiving treatment at the Psychiatric Clinic of the University of Pisa and 55 healthy controls (HCs). The participants' ages ranged from 18 to 65 years. Individuals under the age of 18, those with significant intellectual or language impairments, schizophrenia, a history of substance abuse, severe neurological or medical conditions, or those unable to independently provide written consent or complete psychometric questionnaires were excluded from the study. Diagnosis were made through clinical assessment by trained psychiatrist and confirmed with the SCID-5.

HCs were recruited from medical and paramedical staff and medical students. The absence of psychiatric illness was assessed through clinical assessment by trained personnel and the SCID-5. Inclusion criteria for HCs were age between 18 and 65 years, absence of any psychiatric diagnosis according to DSM-5 criteria, and willingness to provide written informed consent.

Participants were given comprehensive information about the study and had the opportunity to ask questions before signing the informed consent form.

The study adhered to the ethical principles outlined in the Declaration of Helsinki and received approval from the ethical committee of Azienda Ospedaliera Universitaria Pisana (AOUP).
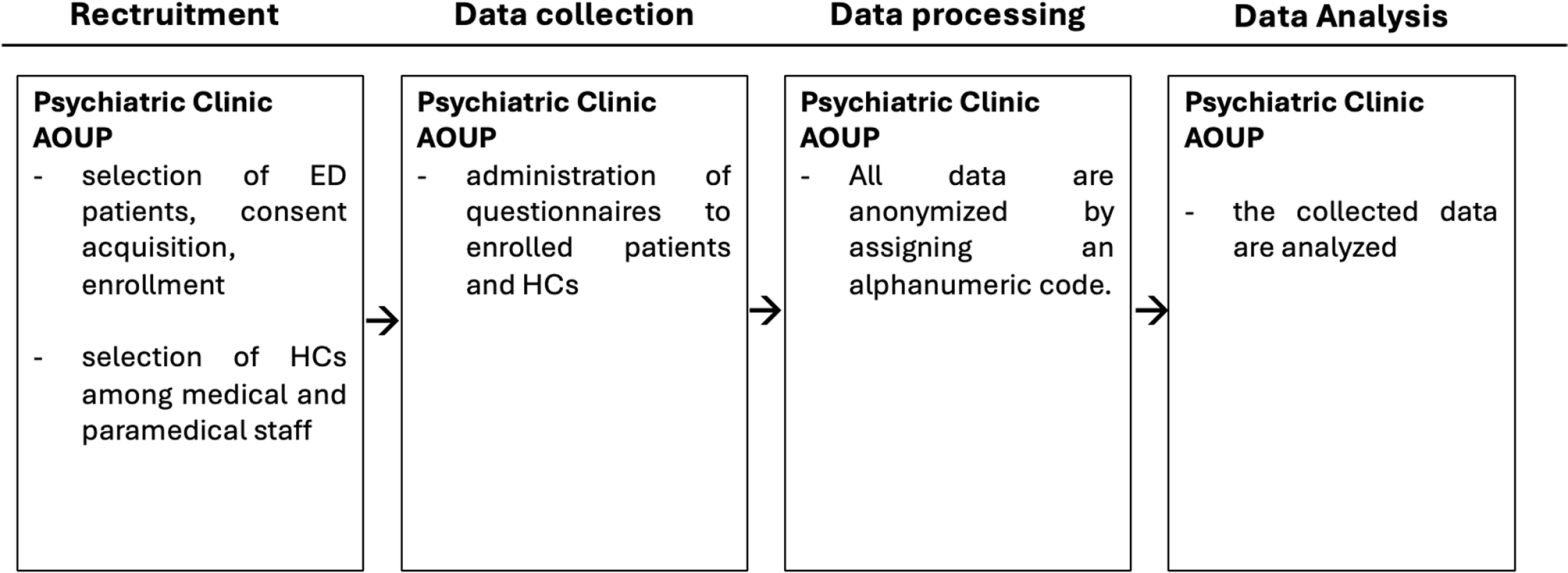


### Measures

#### Camouflaging autistic traits questionnaire CAT-Q

The CAT-Q is questionnaire aimed at assessing camouflaging behaviors associated with the autism spectrum in both clinical and non-clinical populations. This tool consists of 25 items divided into three domains: *assimilation, masking*, and *compensation*, each rated on a seven-point Likert scale. The questionnaire demonstrated outstanding internal consistency, with the Italian version achieving a Cronbach's alpha of 0.904. In addition, it showed strong test–retest reliability and convergent validity when compared to other ASD assessment tools [45, 48]. The questionnaire’s Cronbach's alpha for the current study was 0.959.

#### The eating disorder inventory (EDI-2)

The EDI-2 is a self-report questionnaire that includes 91 items distributed across 11 domains: *drive for thinness, bulimia, body dissatisfaction, ineffectiveness, perfectionism, interpersonal distrust, interoceptive awareness, maturity fears, asceticism,* and *impulsivity*. Respondents provide their answers on a six-point Likert scale. Three of the sub-scales focus specifically on core eating disorder symptoms, such as *Drive for Thinness, Bulimia*, and *Body Dissatisfaction*. The remaining eight sub-scales evaluate psychological traits frequently linked to eating disorders, including *ineffectiveness, maturity fears, social insecurity, perfectionism, interpersonal distrust, impulsivity, interoceptive awareness,* and *asceticism*. This extensive range of sub-scales enables a thorough assessment of both the behavioral and psychological components of eating disorders [49]. The questionnaire demonstrated great internal consistency, with a Cronbach's alpha of 0.904. 78% and 84% when used on clinical samples. The questionnaire’s Cronbach's alpha for the current study was 0.956.

### Statistical analysis

Chi-square test and ANOVA analyses were employed to compare age and gender across diagnostic groups.

Since our sample did not pass the variance homoscedasticity or normality tests, we proceeded to use non-parametric methods.

A Mann–Whitney test was then used to compare scores obtained at the CAT-Q by HCs and EDs patients. Subsequently, a Kruskall–Wallis analysis, followed by Bonferroni post-hoc tests, was performed to compare the scores obtained at the CAT-Q by all five different diagnostic groups. The aim of the analysis was to determine whether there were notable differences in camouflaging behaviors across various EDs.

Afterward, we performed a Spearman correlation analysis with the aim to investigate the presence of significant correlations between CAT-Q and EDI-2 domains and total scores.

Finally, we performed a linear regression analysis using EDI-2 total score as the dependent variable and CAT-Q total score as independent variable to investigate whether the employment of camouflaging strategies was statistically predictive of pathological eating habits.

All statistical analyses were performed with SPSS version 26.0.

## Results

The total sample consisted of 130 participants divided in 70 patients and 55 HCs. Patients were classified into four groups based on their diagnosis:

- 14 patients with anorexia nervosa, restrictive type (AN-R) (*M*: 14.3; *F*: 85.7%; mean age = 36.21 ± 15.43);
- 7 patients with anorexia nervosa, binge-purging type (AN-BP) (*M*: 0%; *F*: 100%; mean age = 22.43 ± 7.85);
- 32 patients with bulimia nervosa (BN) (*M*: 6.3%; *F*: 93.8%; mean age = 33.81 ± 11.70);
- 17 patients with binge eating disorder (BED) (*M*: 11.8%; *F*: 88.2%; mean age = 37.24 ± 15.82).

The four diagnostic groups did not significantly differ in age and gender (see Table 1).

**Table 1:** Age and gender comparison between groups

	HC	AN-R	AN-BP	BN	BED mean ± SD	*F*	*p*
	mean ± SD	mean ± SD	mean ± SD	mean ± SD			
Age	33.96 ± 10.71	36.21 ± 15.43	22.43 ± 7.85	33.81 ± 11.70	37.24 ± 15.82	1.986	0.101
	*n*(%)	*n*(%)	*n*(%)	*n*(%)	*n*(%)	Chi-square	*p*
Gender							
F	47(85.5%)	12(85.7%)	7(100.0%)	30(93.8%)	15(88.2%)	2.430	0.650
M	8 (14.5%)	2(14.3%)	0(0.0%)	2(6.3%)	2(11.8%)		

*Significant for *p* < 0.05

Results from the student *t* test used to compare CAT-Q scores between HCs and patients highlighted that ED patients scored significantly higher than HCs in all CAT-Q domains as well as in its total (Table 2).

**Table 2:** Comparison of CAT-Q scores among HCs, ED patients

	HC mean ± SD, mean rank	ED mean ± SD, mean rank	*U*	*p*
Compensation	14.29 ± 8.85, 39.08	26.68 ± 11.67, 81.79	3240.00	< 0.001*
Masking	15.76 ± 9.42, 34.73	33.48 ± 9.13, 84.64	3425.00	< 0.001*
Assimilation	12.78 ± 6.81, 33.53	30.40 ± 10.75, 85.03	3436.00	< 0.001*
Total	42.84 ± 23.14, 33.52	90.41 ± 26.91, 85.04	3436.50	< 0.001*

*Significant for *p* < 0.05

Results from the subsequent Kruskall–Wallis test showed that HCs scored significantly lower in all CAT-Q domains as well as in its total compared to the other four diagnostic groups, which in turn did not significantly differ from one another (see Table 3).

**Table 3:** Comparison of CAT-Q scores among HCs, AN-R, AN-BP, BN and BED

	HC mean ± SD	AN-R mean ± SD	AN-BP mean ± SD	BN mean ± SD	BED mean ± SD	*H*	*p*
Compens	14.29 ± 88.5	29.50 ± 9.94	36.14 ± 9.97	24.94 ± 11.17	23.76 ± 12.93	48.046	< 0.001*
Masking	15.76 ± 9.42	37.57 ± 7.09	36.43 ± 9.22	32.22 ± 9.08	31.18 ± 9.99	61.519	< 0.001*
Assimil	12.78 ± 6.81	34.93 ± 11.52	32.28 ± 7.11	28.93 ± 10.67	28.47 ± 1.09	64.850	< 0.001*
Total	42.84 ± 23.14	102.00 ± 25.10	104.86 ± 22.09	85.60 ± 25.69	83.41 ± 28.95	66.865	< 0.001*

*AN-R, AN-BP, BN, BED > HCs; significant for *p* < 0.05

Results from the Spearman correlation analysis showed that all CAT-Q domains and total score were significantly positively correlated with all EDI-2 domains, with the only exception of EDI-2 *Interpersonal Distrust* and *Social Insecurity* which did not correlate with CAT-Q *Compensation* and *Masking* domains, and total score (see Table 4). The correlations coefficients ranged from weak to strong. In particular, the strongest correlation were highlighted between EDI-2 *Impulsivity* domain and total score and all CAT-Q domain and total score, and with EDI-2 *Ineffectiveness* and *Asceticism* domains and most CAT-Q domains and total score.

**Table 4:** Spearman correlation coefficients between CAT-Q and EDI-2 domains and total scores in the overall sample

	Compensation	Masking	Assimilation	Total
Drive for thinness	0.470**	0.525**	0.545**	0.559**
Bulimia	0.469**	0.477**	0.459**	0.503**
Body dissatisfaction	0.483**	0.464**	0.425**	0.500**
Ineffectiveness	0.422**	0.570**	0.588**	0.579**
Perfectionism	0.495**	0.547**	0.591**	0.607**
Interpersonal distrust	0.039	0.105	0.207*	0.147
Interoceptive awareness	0.463**	0.548**	0.578**	0.573**
Maturity fears	0.453**	0.491**	0.573**	0.565**
Asceticism	0.586**	0.661**	0.703**	0.706**
Impulsivity	0.607**	0.651**	0.672**	0.700**
Social insecurity	0.052	0.136*	287*	0.187*
Total score	0.577**	0.665**	0.790**	0.701**

*Significant for *p* < 0.05; ** significant for *p* < 0.01

Finally, results from the linear regression analysis performed using EDI-2 total score as dependent variable and CAT-Q total score as independent variable highlighted how the employment of camouflaging strategies was statistically predictive of a higher risk for pathological eating habits (see Table 5).

**Table 5:** Linear regression analyses with EDI-2 total score as a dependent variable and CAT-Q total score as independent variable in the overall group

	*B* (S.E.)	Beta	*t*	*p*
Constant	3.302 (6.067)		0.544	0.587
CAT-Q total score	0.917 (0.079)	0.728	11.678	< 0.001*

*R*^2^: 530; Adjusted *R*^2^: 0.526

## Discussion

The aim of this study was to investigate the prevalence and correlates of camouflaging strategies related to autistic features among patients with ED.

While the concept of social camouflaging has been extensively explored in the autism literature, its investigation within ED populations remains relatively limited. However, the idea of camouflaging can be conceptually situated within the broader framework of maladaptive coping strategies, which have long been implicated in the development and maintenance of EDs. Individuals with EDs frequently adopt maladaptive coping mechanisms, such as dietary restriction, binge eating, or excessive control over food, as a way to manage emotional distress, interpersonal challenges, and internalized social pressures [50, 51]. In this context, social camouflaging may represent another form of coping, particularly in individuals with elevated autistic traits who may feel compelled to mask social difficulties to avoid rejection or fit social norms. Framing camouflaging within this broader coping model helps clarify its relevance to ED psychopathology and aligns with prior findings that emotion regulation and social functioning play critical roles in both conditions.

Our findings revealed that individuals with ED scored significantly higher in all domains of the CAT-Q, as well as in the total score, compared to HCs, with no significant differences between the AN-R, AN-BP, BN and BED. These data are consistent with a growing body of research suggesting that, in some cases, ED may represent an alternative expression of ASD in females, in which the restricted interests and repetitive behaviors typical of autism spectrum would be focused on food and diet [9, 10, 36, 52]. In fact, females with ASD are often unrecognized or late-diagnosed, because they do not fit the stereotypical conceptualization of autism that is based primarily on male presentations [53].

In this framework, the relationship between AN and autism spectrum has been a topic of interest for many years, with foundational work by Gillberg in 1983 [11], and subsequent studies reinforcing the connection. These studies have consistently shown striking similarities in the neurobiological and neuropsychological structures of AN and ASD, as well as shared challenges with empathy, social interaction, and emotional reciprocity [9, 11, 12, 23, 52].

In more recent years, research has expanded to explore the relationship between autism and other eating disorders. For example, a study from 2018 found that individuals with AN, BN, and BED exhibited more pronounced autistic traits compared to HCs [54]. Another study in 2021 demonstrated that individuals with ED showed higher levels of autistic traits than HCs, while individuals with high-functioning autism had significantly higher scores for eating disorders compared to HCs [55].

Another key characteristic of the female autism phenotype is "camouflaging," which refers to the ability to mask or suppress autistic traits by imitating the behaviors of neurotypical individuals [39, 42, 56]. Indeed, in numerous interviews, women who were later diagnosed with ASD have described the conscious effort they put into learning and using "neurotypical" social skills. Some even likened their experience to "wearing a mask" to fit-in with societal expectations [57]. This phenomenon can be attributed to the fact that women with ASD typically show stronger social motivation and a greater capacity to form social connections compared to their male counterparts [58]. Women are often more driven to adjust to social norms, despite the challenges they may face in understanding social cues and interactions. Thus, the high levels of camouflaging observed in individuals with EDs align with the evidence showing that individuals with eating disorders, are more likely to present elevated autistic traits or even full-blown ASD, and in turn employ social camouflaging as a coping strategy for adjust to their social difficulties and mask their autism spectrum, driven by the greater social motivation observed in the female autism phenotype [59].

Moreover, although lacking of a significant value, patients with AN appeared to show higher CAT-Q scores than those with BN and BED. The lack of statistical significance should be interpreted cautiously given the limited number of participants due to the division of the AN sample in two separate subgroups (R and BN). Future studies with larger and more balanced subgroup samples are needed to clarify whether individuals with AN exhibit distinct patterns of social camouflaging.

Moreover, our results highlighted significant positive correlations between all CAT-Q domains and their total scores and all EDI-2 domains, with the only exception of *Interpersonal Distrust* and *Social Insecurity*. This lack of correlations can be explained by the fact that camouflage and compensation are often used for a specific purpose, such as to fit-in or avoid social rejection [40]. The person does not necessarily feel distrust toward others but is instead motivated to present themselves in a socially acceptable way. It is, therefore, a strategic approach to social situations rather than a response based on distrust. Furthermore, compensation is a coping mechanism that allows people to function better in social contexts, where they might otherwise feel overwhelmed. Although it can mask internal difficulties, it can also create a sense of temporary relief, as it allows the person to avoid perceived failure or rejection in social interactions [42, 60]. This sense of control can counteract feelings of social insecurity, as the person feels able to handle the situation.

The strongest correlation emerged between CAT-Q and EDI-2 total score, supporting the evidence of a tendency of more severe eating disorder symptoms when it is associated with the presence of autistic traits [9, 10]. Moreover, some of the strongest correlations involved camouflaging and EDI-2 *Impulsivity, Ineffectiveness* and *Asceticism* domains. Even though the literature on the topic is still scant, it is possible to formulate some assumptions on the reasons behind the finding of such correlations. The relationship between impulsivity and camouflaging may arise from the emotional strain associated with constantly using strategies to hide one's social difficulties. Individuals who engage in camouflaging often focus intensely on masking their social challenges to fit-in or meet social expectations. Over time, this constant effort can become emotionally taxing [39, 40, 60]. In moments of emotional dysregulation or when control slips, this pressure might manifest in strong emotional reactions or impulsive behaviors, in line with previous researches that reported how camouflaging may predict emotional dysregulation in subjects with autism spectrum conditions and that emotional regulation challenges may mediate the relationship between social camouflaging and depression or anxiety symptoms [61, 62]. This dynamic is particularly plausible when we consider the difficulties people on the autism spectrum often face in managing their emotions. Indeed, research has shown that individuals with autism, or those displaying autistic traits, may be more prone to emotional outbursts or find it harder to regulate their emotions, which could exacerbate the challenges of camouflaging [63–65].

Even asceticism, conceptualized as the tendency to sacrifice one’s own needs, may be intertwined with camouflaging behaviors. Indeed, it is plausible that when a person focuses on fitting into social settings by masking their challenges, they may prioritize these external social goals over their own internal emotional (and nutritional) needs. In this process, they may dismiss or suppress their emotions and desires, seeing this denial as a way to perform better socially [40, 56, 66, 67]. In this sense, asceticism may become a coping strategy: the individual might convince themselves that sacrificing their emotional well-being is necessary to achieve socially acceptable interactions. This can lead to a distorted view, where the individual believes that by sacrificing their emotional needs, they are improving their social performance, even though this neglect could negatively affect their well-being in the long run [40, 66].

In relation to the Ineffectiveness domain, we can hypothesize that the difficulties individuals experience in social interactions—along with the need to constantly engage in camouflaging—can contribute to feelings of inauthenticity or failure [60, 66]. When a person perceives that social connections are only successful, because they are performing a learned behavior, rather than being themselves, they may begin to feel disconnected from their true identity. This can lead to a diminished sense of self-worth, as they may view their social interactions as mere performances rather than genuine exchanges. This feeling of being “inauthentic” or “fake” could erode the person’s sense of self-efficacy, diminishing their belief in their ability to form meaningful connections [67–69]. Over time, this can contribute to lower self-esteem, as they internalize the idea that their social success is contingent on their ability to mask or perform, rather than being rooted in their true self [68, 69].

Finally, the linear regression results revealed that the total score on the CAT-Q was a significant predictor of increased eating disorder symptoms as measured by EDI-2. These findings align with previous reports, which suggest that eating disorders may be linked to camouflaging behaviors [47, 52, 70]. In particular, a study from Bradley et al. reported a predictive role of social camouflaging, as measured by the CAT-Q, on ED symptomatology in autistic adults [47], hypothesizing that ED symptoms may be favored by camouflaging tendencies, as a way to cope with social demands. A particularly interesting theme that has emerged from studies of autistic individuals with EDs is the critical role of social identity in the development and persistence of these disorders [52, 71]. In fact, some have proposed that eating disorders, especially AN, might emerge from identity struggles related to having high autistic traits. In this framework, for many individuals on the spectrum, an eating disorder may offer a sense of identity or a group to belong to, or it may serve as a coping strategy when they struggle to adapt to societal expectations [52]. Eventually, it is also possible that, to fit-in with other people, subjects in the autism spectrum may focus on adopt socially accepted interest and standards, such as food, diet or body weight, pursuing them with the pervasive and inflexible modality typical of the autism spectrum, ultimately increasing the risk to develop a ED. These findings globally contribute to the broader body of research suggesting that camouflaging behaviors are closely linked to poor mental health outcomes, including eating disorder symptoms [47, 67, 72].

The present work should be considered in the context of several limitations. First, the study’s cross-sectional design limits our ability to draw conclusions about cause-and-effect relationships or the temporal progression of the variables being examined. In addition, the reliance on self-report psychometric questionnaires to assess participants may lead to either over- or under-estimation of symptoms, depending on individuals' subjective perceptions. Furthermore, the relatively small sample size reduces the ability to generalize the results, making it challenging to apply these findings to larger or more diverse populations. Moreover, the small numerosity of some diagnostic groups may challenge the assumptions of certain statistical tests. For this reason, we considered alternative approaches (e.g., combining subgroups, such as AN-R and AN-BP), and while we retained the original groupings for transparency and consistency with diagnostic distinctions, we recognize that this may limit the robustness of some comparisons. Future studies with larger and more balanced samples are needed to confirm these findings. Finally, the sample included a relatively small number of male participants, which may limit the generalizability of our findings across genders. Given that social camouflaging behaviors and the expression of autistic traits can differ significantly between males and females, the current results may be more reflective of the female phenotype. Future studies with more balanced gender representation are needed to better understand gender-specific patterns in the relationship between camouflaging and eating disorder symptomatology.

## Conclusions

This study found that individuals with EDs exhibited significantly higher levels of social camouflaging compared to HCs. The results showed no significant differences across the different subtypes of eating disorders, suggesting a general trend of heightened camouflaging behaviors in individuals with ED. We also observed significant positive correlations between camouflaging behaviors and various eating disorder symptom domains. While our regression analysis showed that camouflaging behaviors were statistically associated with greater severity of eating disorder symptoms, this finding should be interpreted with caution due to the cross-sectional design. As such, the observed relationship may suggest, but cannot confirm, a possible contributory role of camouflaging in disordered eating. These findings support the evidence that the use of social camouflaging as a coping mechanism would be associated with eating disorder symptomatology. Overall, this study highlights the complex interplay between autistic traits and eating disorder symptoms, emphasizing the need for further research into this relationship. Longitudinal studies are needed to better understand the directionality and potential causal links between these variables.

Moreover, considering the hypothesis that maladaptive coping strategies may play a central role in the development of EDs, it would be important for future research to also explore their impact on non-clinical disordered eating behaviors. Understanding how coping mechanisms contribute to subthreshold or emerging eating difficulties, such as emotional eating, food avoidance, or body dissatisfaction, could offer valuable insights for early intervention. This is particularly relevant given that, if left unaddressed, such behaviors often intensify over time and may progress into full-syndrome eating disorders.

### Strength and limits

This study offers valuable insights into the relationship between social camouflaging and eating disorder symptoms, highlighting the role of autistic traits in the clinical presentation of EDs. Its strengths include the use of validated assessment tools (CAT-Q and EDI-2), a well-defined clinical and control sample, and robust statistical analyses that support the reliability of the findings. However, the study's cross-sectional design limits the ability to infer causal relationships, and the relatively small sample size may restrict generalizability. In addition, reliance on self-report measures introduces the potential for response bias. Future longitudinal and experimental studies are needed to clarify the directionality and mechanisms underlying these associations.

### What is already known on this subject?

Prior research has shown a notable overlap between autistic traits and EDs, particularly in females, with some studies suggesting that disordered eating may represent a gender-specific manifestation of autism. While social camouflaging has been linked to psychological distress in individuals with autism, its role in the context of EDs had not been systematically explored. This study was needed to investigate whether camouflaging behaviors contribute to the severity of eating disorder symptoms in individuals with EDs.

### What this study adds?

This study demonstrates that individuals with EDs exhibit significantly higher levels of social camouflaging compared to HCs, regardless of ED subtype. It also shows that camouflaging behaviors are positively correlated with the severity of eating disorder symptoms and can predict pathological eating patterns. These findings highlight the importance of assessing autistic traits and camouflaging strategies in ED populations, with implications for more tailored diagnostic and therapeutic approaches.

## Data Availability

No data sets were generated or analyzed during the current study.

## References

[1] Treasure J, Duarte TA, Schmidt U (2020) Eating disorders. Lancet 395(10227):899–911. 10.1016/S0140-6736(20)30059-3 32171414 10.1016/S0140-6736(20)30059-3

[2] Gibson D, Workman C, Mehler PS (2019) Medical complications of anorexia nervosa and bulimia nervosa. Psychiatr Clin North Am 42(2):263–274. 10.1016/j.psc.2019.01.009 31046928 10.1016/j.psc.2019.01.009

[3] American Psychiatric Association (2022) Diagnostic and statistical manual of mental disorders; DSM-V-TR. American Psychiatric Association, Washington, DC, USA

[4] World Health Organization (2019) International classification of diseases for mortality and morbidity statistics (11th Revision). https://icd.who.int/

[5] Yu Z, Muehleman V (2023) Eating disorders and metabolic diseases. Int J Environ Res Public Health 20(3):2446. 10.3390/ijerph20032446 36767812 10.3390/ijerph20032446 PMC9916228

[6] Bhattacharya A, DeFilipp L, Timko CA (2020) Feeding and eating disorders. Handb Clin Neurol 175:387–403. 10.1016/B978-0-444-64123-6.00026-6 33008539 10.1016/B978-0-444-64123-6.00026-6

[7] Abu-Akel A, Allison C, Baron-Cohen S, Heinke D (2019) The distribution of autistic traits across the autism spectrum: evidence for discontinuous dimensional subpopulations underlying the autism continuum. Mol Autism 10:24. 10.1186/s13229-019-0275-3 31149329 10.1186/s13229-019-0275-3 PMC6537408

[8] Gillberg C (1985) Autism and anorexia nervosa: related conditions? Nord Psykiatr Tidsskr 39(4):307–312. 10.1192/bjp.142.4.428b

[9] Boltri M, Sapuppo W (2021) Anorexia nervosa and autism spectrum disorder: a systematic review. Psychiatry Res 306:114271. 10.1016/j.psychres.2021.114271 34798485 10.1016/j.psychres.2021.114271

[10] Schröder SS, Danner UN, Spek AA, van Elburg AA (2023) Exploring the intersection of autism spectrum disorder and eating disorders: understanding the unique challenges and treatment considerations for autistic women with eating disorders. Curr Opin Psychiatry 36(6):419–426. 10.1097/YCO.0000000000000894 37781983 10.1097/YCO.0000000000000894

[11] Parsons MA (2023) Autism diagnosis in females by eating disorder professionals. J Eat Disord 11(1):73. 10.1186/s40337-023-00785-0 37170136 10.1186/s40337-023-00785-0 PMC10173598

[12] Huke V, Turk J, Saeidi S, Kent A, Morgan JF (2013) Autism spectrum disorders in eating disorder populations: a systematic review. Eur Eat Disord Rev 21(5):345–351. 10.1002/erv.2244 23900859 10.1002/erv.2244

[13] Gillberg C (1983) Are autism and anorexia nervosa related? Br J Psychiatry 142(4):428. 10.1192/bjp.142.4.428b 6850191 10.1192/bjp.142.4.428b

[14] Westwood H, Tchanturia K (2017) Autism spectrum disorder in anorexia nervosa: an updated literature review. Curr Psychiatry Rep 19(7):41. 10.1007/s11920-017-0791-9 28540593 10.1007/s11920-017-0791-9 PMC5443871

[15] Westwood H, Mandy W, Tchanturia K (2017) Clinical evaluation of autistic symptoms in women with anorexia nervosa. Mol Autism 8:12 28331571 10.1186/s13229-017-0128-x PMC5356303

[16] Westwood H, Eisler I, Mandy W, Leppanen J, Treasure J, Tchanturia K (2016) Using the autism-spectrum quotient to measure autistic traits in anorexia nervosa: a systematic review and meta-analysis. J Autism Dev Disord 46(3):964–977. 10.1007/s10803-015-2641-0 26542816 10.1007/s10803-015-2641-0 PMC4746216

[17] Baron-Cohen S, Jaffa T, Davies S, Auyeung B, Allison C, Wheelwright S (2013) Do girls with anorexia nervosa have elevated autistic traits? Mol Autism 4(1):24. 10.1186/2040-2392-4-24 23915495 10.1186/2040-2392-4-24 PMC3735388

[18] Tchanturia K, Davies H, Harrison A, Fox JR, Treasure J, Schmidt U (2012) Altered social hedonic processing in eating disorders. Int J Eat Disord 45(8):962–969. 10.1002/eat.22032 22693046 10.1002/eat.22032

[19] Hambrook D, Brown G, Tchanturia K (2012) Emotional intelligence in anorexia nervosa: is anxiety a missing piece of the puzzle? Psychiatry Res 200(1):12–19. 10.1016/j.psychres.2012.05.017 22703722 10.1016/j.psychres.2012.05.017

[20] Davies H, Schmidt U, Stahl D, Tchanturia K (2011) Evoked facial emotional expression and emotional experience in people with anorexia nervosa. Int J Eat Disord 44(6):531–539. 10.1002/eat.20852 20957704 10.1002/eat.20852

[21] Tchanturia K, Happé F, Godley J, Treasure J, Bara-Carril N, Schmidt U (2004) ‘Theory of mind’in anorexia nervosa. Europ Eat Disord Rev 12(6):361–366. 10.1002/erv.608

[22] Carpita B, Nardi B, Pronestì C, Cerofolini G, Filidei M, Bonelli C, Massimetti G, Cremone IM, Pini S, Dell’Osso L (2025) The mediating role of social camouflaging on the relationship between autistic traits and orthorexic symptoms. Brain Sci 15(5):503. 10.3390/brainsci15050503 40426674 10.3390/brainsci15050503 PMC12109957

[23] Kerr-Gaffney J, Mason L, Jones E, Hayward H, Ahmad J, Harrison A, Loth E, Murphy D, Tchanturia K (2020) Emotion recognition abilities in adults with anorexia nervosa are associated with autistic traits. J Clin Med 9(4):1057. 10.3390/jcm9041057 32276387 10.3390/jcm9041057 PMC7230901

[24] Kerr-Gaffney J, Harrison A, Tchanturia K (2020) Autism spectrum disorder traits are associated with empathic abilities in adults with anorexia nervosa. J Affect Disord 266:273–281. 10.1016/j.jad.2020.01.169 32056888 10.1016/j.jad.2020.01.169

[25] Courty A, Maria AS, Lalanne C, Ringuenet D, Vindreau C, Chevallier C, Pouga L, Pinabel F, Philippe A, Adrien JL, Barry C, Berthoz S (2013) Levels of autistic traits in anorexia nervosa: a comparative psychometric study. BMC Psychiatry 13:222. 10.1186/1471-244X-13-222 24015680 10.1186/1471-244X-13-222 PMC3848448

[26] Zhou ZC, McAdam DB, Donnelly DR (2018) Endophenotypes: a conceptual link between anorexia nervosa and autism spectrum disorder. Res Dev Disabil 82:153–165. 10.1016/j.ridd.2017.11.008 29239739 10.1016/j.ridd.2017.11.008

[27] Vagni D, Moscone D, Travaglione S, Cotugno A (2016) Using the Ritvo Autism Asperger Diagnostic Scale-Revised (RAADS-R) disentangle the heterogeneity of autistic traits in an Italian eating disorder population. Res Autism Spectr Disord 32:143–155. 10.1016/j.rasd.2016.10.002

[28] Dinkler L, Rydberg Dobrescu S, Råstam M, Gillberg IC, Gillberg C, Wentz E, Hadjikhani N (2019) Visual scanning during emotion recognition in long-term recovered anorexia nervosa: an eye-tracking study. Int J Eat Disord 52(6):691–700. 10.1002/eat.23066 30828832 10.1002/eat.23066

[29] Karjalainen L, Råstam M, Paulson-Karlsson G, Wentz E (2019) Do autism spectrum disorder and anorexia nervosa have some eating disturbances in common? Eur Child Adolesc Psychiatry 28(1):69–78. 10.1007/s00787-018-1188-y 29974245 10.1007/s00787-018-1188-y PMC6349794

[30] Gabriel T, Paul S, Berger A, Massoubre C (2019) Anorexia nervosa and autism spectrum disorders: future hopes linked to mucosal immunity. NeuroImmunoModulation 26(6):265–275. 10.1159/000502997 31715599 10.1159/000502997

[31] Nazar BP, Peynenburg V, Rhind C, Hibbs R, Schmidt U, Gowers S (2018) An examination of the clinical outcomes of adolescents and young adults with broad autism spectrum traits and autism spectrum disorder and anorexia nervosa: a multi centre study. Int J Eat Disord 51(2):174–179. 10.1002/eat.22823.29331075 29331075 10.1002/eat.22823

[32] Brown CM, Stokes MA (2020) Intersection of eating disorders and the female profile of autism. Psychiatr Clin North Am 43(4):735–743. 10.1016/j.psc.2020.08.009 33127005 10.1016/j.psc.2020.08.009

[33] Babb C, Brede J, Jones CRG, Elliott M, Zanker C, Tchanturia K, Serpell L, Mandy W, Fox JRE (2021) “It’s not that they don’t want to access the support … it’s the impact of the autism”: the experience of eating disorder services from the perspective of autistic women, parents and healthcare professionals. Autism 25(5):1409–1421. 10.1177/1362361321991257 33588579 10.1177/1362361321991257 PMC8264634

[34] Kinnaird E, Norton C, Stewart C, Tchanturia K (2019) Same behaviours, different reasons: What do patients with co-occurring anorexia and autism want from treatment? Int Rev Psychiatry 31(4):308–317. 10.1080/09540261.2018.1531831 30821179 10.1080/09540261.2018.1531831

[35] Christiansen GB, Petersen LV, Chatwin H, Yilmaz Z, Schendel D, Bulik CM, Grove J, Brikell I, Semark BD, Holde K, Abdulkadir M, Hübel C, Albiñana C, Vilhjálmsson BJ, Børglum AD, Demontis D, Mortensen PB, Larsen JT (2025) The role of co-occurring conditions and genetics in the associations of eating disorders with attention-deficit/hyperactivity disorder and autism spectrum disorder. Mol Psychiatry 30(5):2127–2136. 10.1038/s41380-024-02825-w 39543370 10.1038/s41380-024-02825-w PMC12014370

[36] Carpita B, Muti D, Cremone IM, Fagiolini A, Dell’Osso L (2022) Eating disorders and autism spectrum: links and risks. CNS Spectr 27(3):272–280. 10.1017/S1092852920002011 33161925 10.1017/S1092852920002011

[37] Rastam M (2008) Eating disturbances in autism spectrum disorders with focus on adolescent and adult years. Clin Neuropsychiatry 5(1):31–42

[38] Bayoumi SC, Halkett A, Miller M, Hinshaw SP (2025) Food selectivity and eating difficulties in adults with autism and/or ADHD. Autism 29(6):1497–1509. 10.1177/13623613251314223 39996584 10.1177/13623613251314223 PMC12089672

[39] Dell’Osso L, Lorenzi P, Carpita B (2021) Camouflaging: psychopathological meanings and clinical relevance in autism spectrum conditions. CNS Spectr 26(5):437–439. 10.1017/S1092852920001467 32450944 10.1017/S1092852920001467

[40] Hull L, Petrides KV, Allison C, Smith P, Baron-Cohen S, Lai MC, Mandy W (2017) “Putting on my best normal”: Social camouflaging in adults with autism spectrum conditions. J Autism Dev Disord 47(8):2519–2534. 10.1007/s10803-017-3166-5 28527095 10.1007/s10803-017-3166-5 PMC5509825

[41] Lai MC, Lombardo MV, Ruigrok AN, Chakrabarti B, Auyeung B, Szatmari P, Happé F, Baron-Cohen S, MRC AIMS (2017) Consortium quantifying and exploring camouflaging in men and women with autism. Autism 21(6):690–702. 10.1177/1362361316671012 27899710 10.1177/1362361316671012 PMC5536256

[42] Cook J, Hull L, Crane L, Mandy W (2021) Camouflaging in autism: a systematic review. Clin Psychol Rev 89:102080. 10.1016/j.cpr.2021.102080 34563942 10.1016/j.cpr.2021.102080

[43] McQuaid GA, Lee NR, Wallace GL (2022) Camouflaging in autism spectrum disorder: examining the roles of sex, gender identity, and diagnostic timing. Autism 26(2):552–559. 10.1177/13623613211042131 34420418 10.1177/13623613211042131

[44] Loomes R, Hull L, Mandy WPL (2017) What is the male-to-female ratio in autism spectrum disorder? A systematic review and meta-analysis. J Am Acad Child Adolesc Psychiatry 56(6):466–474. 10.1016/j.jaac.2017.03.013 28545751 10.1016/j.jaac.2017.03.013

[45] Dell’Osso L, Cremone IM, Muti D, Massimetti G, Lorenzi P, Carmassi C, Carpita B (2022) Validation of the Italian version of the Camouflaging Autistic Traits Questionnaire (CAT-Q) in a university population. Compr Psychiatry 114:152295. 10.1016/j.comppsych.2022.152295 35042086 10.1016/j.comppsych.2022.152295

[46] Dell’Osso L, Cremone IM, Chiarantini I, Arone A, Massimetti G, Carmassi C, Carpita B (2022) Autistic traits and camouflaging behaviors: a cross-sectional investigation in a university student population. CNS Spectr 27(6):740–746. 10.1017/S1092852921000808 34505557 10.1017/S1092852921000808

[47] Bradley S, Moore F, Duffy F, Clark L, Suratwala T, Knightsmith P, Gillespie-Smith K (2024) Camouflaging, not sensory processing or autistic identity, predicts eating disorder symptoms in autistic adults. Autism 28(11):2858–2868. 10.1177/13623613241245749 38634458 10.1177/13623613241245749 PMC11497744

[48] Hull L, Mandy W, Lai MC, Baron-Cohen S, Allison C, Smith P, Petrides KV (2019) Development and validation of the Camouflaging Autistic Traits Questionnaire (CAT-Q). J Autism Dev Disord 49:819–833. 10.1007/s10803-018-3792-6 30361940 10.1007/s10803-018-3792-6 PMC6394586

[49] Garner DM (1991) Eating disorder inventory-2. Psychological Assessment Resources, Odessa, Fl

[50] Estévez A, Momeñe J, Macía L, Iruarrizaga I, Olave L, Aonso-Diego G (2024) The mediating effect of coping strategies and emotion regulation in the relationship between impulsivity, metacognition, and eating disorders. Nutrients 16(12):1884. 10.3390/nu16121884 38931239 10.3390/nu16121884 PMC11206882

[51] Sulkowski ML, Dempsey J, Dempsey AG (2011) Effects of stress and coping on binge eating in female college students. Eat Behav 12(3):188–191. 10.1016/j.eatbeh.2011.04.006 21741016 10.1016/j.eatbeh.2011.04.006

[52] Brede J, Babb C, Jones C, Elliott M, Zanker C, Tchanturia K, Mandy W (2020) ‘For me, the anorexia is just a symptom, and the cause is the autism’: Investigating restrictive eating disorders in autistic women. J Autism Dev Disord 50(12):4280–4296. 10.1007/s10803-020-04479-3 32274604 10.1007/s10803-020-04479-3 PMC7677288

[53] Lai MC, Baron-Cohen S (2015) Identifying the lost generation of adults with autism spectrum conditions. Lancet Psychiatry 2(11):1013–1027. 10.1016/S2215-0366(15)00277-1 26544750 10.1016/S2215-0366(15)00277-1

[54] Dell’Osso L, Carpita B, Gesi C, Cremone IM, Corsi M, Massimetti E, Muti D, Calderani E, Castellini G, Luciano M, Ricca V, Carmassi C, Maj M (2018) Subthreshold autism spectrum disorder in patients with eating disorders. Compr Psychiatry 81:66–72. 10.1016/j.comppsych.2017.11.007 29268154 10.1016/j.comppsych.2017.11.007

[55] Nisticò V, Faggioli R, Bertelli S, Priori A, Gambini O, Demartini B (2022) Eating disturbances in eating disorders and in high-functioning autism spectrum disorders: a preliminary study. Eat Weight Disord 27(4):1555–1561. 10.1007/s40519-021-01225-1 34264484 10.1007/s40519-021-01225-1

[56] Willey LH (2014) Pretending to be normal: living with Asperger's syndrome (Autism Spectrum Disorder) Expanded Edition. Jessica Kingsley Publishers

[57] Baldwin S, Costley D (2016) The experiences and needs of female adults with high-functioning autism spectrum disorder. Autism 20(4):483–495. 10.1177/1362361315590805 26111537 10.1177/1362361315590805

[58] Sedgewick F, Hill V, Yates R, Pickering L, Pellicano E (2016) Gender differences in the social motivation and friendship experiences of autistic and non-autistic adolescents. J Autism Dev Disord 46(4):1297–1306. 10.1007/s10803-015-2669-1 26695137 10.1007/s10803-015-2669-1 PMC4786616

[59] Green RM, Travers AM, Howe Y, McDougle CJ (2019) Women and autism spectrum disorder: diagnosis and implications for treatment of adolescents and adults. Curr Psychiatry Rep 21(4):22. 10.1007/s11920-019-1006-3 30852705 10.1007/s11920-019-1006-3

[60] Cremone IM, Carpita B, Nardi B, Casagrande D, Stagnari R, Amatori G, Dell’Osso L (2023) Measuring social camouflaging in individuals with high functioning autism: a literature review. Brain Sci 13(3):469. 10.3390/brainsci13030469 36979279 10.3390/brainsci13030469 PMC10046375

[61] Bemmouna D, Lagzouli A, Weiner L (2023) The biosocial correlates and predictors of emotion dysregulation in autistic adults compared to borderline personality disorder and nonclinical controls. Mol Autism 14(1):47. 10.1186/s13229-023-00580-3 38110995 10.1186/s13229-023-00580-3 PMC10726572

[62] McQuaid GA, Sadowski LY, Lee NR, Wallace GL (2024) An examination of perceived stress and emotion regulation challenges as mediators of associations between camouflaging and internalizing symptomatology. Autism Adulthood 6(3):345–361. 10.1089/aut.2022.0121 39371362 10.1089/aut.2022.0121 PMC11447396

[63] Dell’Osso L, Massoni L, Battaglini S, De Felice C, Nardi B, Amatori G, Cremone IM, Carpita B (2023) Emotional dysregulation as a part of the autism spectrum continuum: a literature review from late childhood to adulthood. Front Psychiatry 14:1234518. 10.3389/fpsyt.2023.1234518 37791135 10.3389/fpsyt.2023.1234518 PMC10544895

[64] Restoy D, Oriol-Escudé M, Alonzo-Castillo T, Magán-Maganto M, Canal-Bedia R, Díez-Villoria E, Gisbert-Gustemps L, Setién-Ramos I, Martínez-Ramírez M, Ramos-Quiroga JA, Lugo-Marín J (2024) Emotion regulation and emotion dysregulation in children and adolescents with Autism Spectrum Disorder: a meta-analysis of evaluation and intervention studies. Clin Psychol Rev 109:102410. 10.1016/j.cpr.2024.102410 38401510 10.1016/j.cpr.2024.102410

[65] Hervas A (2017) Desregulacion emocional y trastornos del espectro autista [Emotional dysregulation and autism spectrum disorders]. Rev Neurol 64(s01):S17–S25 28256682

[66] Bernardin CJ, Mason E, Lewis T, Kanne S (2021) “You must become a chameleon to survive”: adolescent experiences of camouflaging. J Autism Dev Disord 51(12):4422–4435. 10.1007/s10803-021-04912-1 33590426 10.1007/s10803-021-04912-1

[67] Bargiela S, Steward R, Mandy W (2016) The experiences of late-diagnosed women with autism spectrum conditions: an investigation of the female autism phenotype. J Autism Dev Disord 46:3281–3294. 10.1007/s10803-016-2872-8 27457364 10.1007/s10803-016-2872-8 PMC5040731

[68] Tierney S, Burns J, Kilbey E (2016) Coping strategies of girls on the autistic spectrum. Res Autism Spectr Disord 23:73–83. 10.1016/j.rasd.2015.11.013

[69] Cage E, Di Monaco J, Newell V (2018) Experiences of autism acceptance and mental health in autistic adults. J Autism Dev Disord 48:473–484. 10.1007/s10803-017-3342-7 29071566 10.1007/s10803-017-3342-7 PMC5807490

[70] Adamson J, Kinnaird E, Glennon D, Oakley M, Tchanturia K (2020) Carers’ views on autism and eating disorders comorbidity: qualitative study. BJPsych Open 6(3):e51. 10.1192/bjo.2020.36 32419683 10.1192/bjo.2020.36 PMC7331083

[71] Dandil Y, Baillie C, Tchanturia K (2019) Cognitive remediation therapy as a feasible treatment for a young person with anorexia nervosa and autism spectrum disorder comorbidity: a case study. Clin Case Stud 19(2):115–132. 10.1177/1534650119890425

[72] Beck JS, Lundwall RA, Gabrielsen T, Cox JC, South M (2020) Looking good but feeling bad: ‘camouflaging’ behaviors and mental health in women with autistic traits. Autism 24(4):809–821. 10.1177/1362361320912147 32429817 10.1177/1362361320912147

